# A rare case of perforated jejunal diverticula of an uncommon origin

**DOI:** 10.1002/ccr3.7206

**Published:** 2023-04-12

**Authors:** Aleeza Jawed, Areesha Jawed, Sandhya Kumari, Omer Ahmed Shaikh, Abdulqadir J. Nashwan

**Affiliations:** ^1^ Department of Medicine Ziauddin University Karachi Pakistan; ^2^ Department of Medicine Dow University of Health Sciences Karachi Pakistan; ^3^ Hamad Medical Corporation Doha Qatar

**Keywords:** duodenojejunal junction, ileal edema, ileostomy, perforated jejunal diverticula

## Abstract

Jejunal diverticula are often asymptomatic and rare, so they can go unnoticed until serious complications like obstruction, bleeding, perforation, volvulus, or diverticulitis occur. Elderly people over 60 are more likely to have this condition.

## Graphical abstract

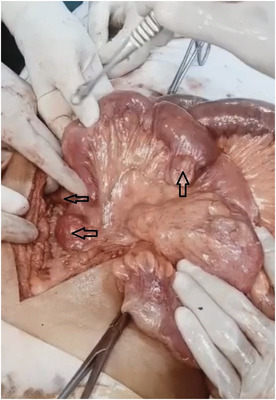

## 1 INTRODUCTION

We present a case of a 75‐year‐old male who presented with constipation, nausea, and vomiting for 4 days. Two diverticula were discovered 1 and 2 feet away from the duodenojejunal (DJ) junction. Due to excessive ileal edema, ileal resection, and a double barrel ileostomy was performed.

Small bowel diverticula are a rare disease, with a reported incidence of 0.3%–1.3% at autopsies and 2.0%–2.3% radiographically.^1^ 61% of small bowel diverticula occur in the jejunum.^2^ These diverticula, which are often many and located along the mesenteric border of the colon, are produced by herniation of the mucosa and submucosa at loci where blood vessels have pierced the gut wall.^2^ Diverticula are more widespread in men than in women and become more prevalent with age, peaking in the sixth and seventh decades of age.^3^ In addition to blood vessel penetration, there are several other postulated mechanisms for the pathophysiology of the disease. It is believed to occur due to a combination of intestinal dyskinesia caused by abnormalities of the smooth muscle and myenteric plexus, and irregular intestinal contractions that cause an increased segmental intraluminal pressure.^4^ Mortality rate ranges from 0% to 5%, but can increase to up to 40% in cases of perforation.^5^

## 2 CASE REPORT

A 75‐year‐old male with no known comorbidities arrived at the emergency department complaining of constipation, nausea, and vomiting for the past 3–4 days. The patient was in his usual state of health when he experienced constipation. He has not passed flatulence for the past 3–4 days. The patient also complained of localized pain in the lower abdomen, which was gradual in onset, dragging in character, and non‐radiating. The patient had 3–4 episodes of vomiting, which was non‐projectile and did not contain any blood or mucous. He had a prior history of on and off constipation.

**FIGURE 1:**
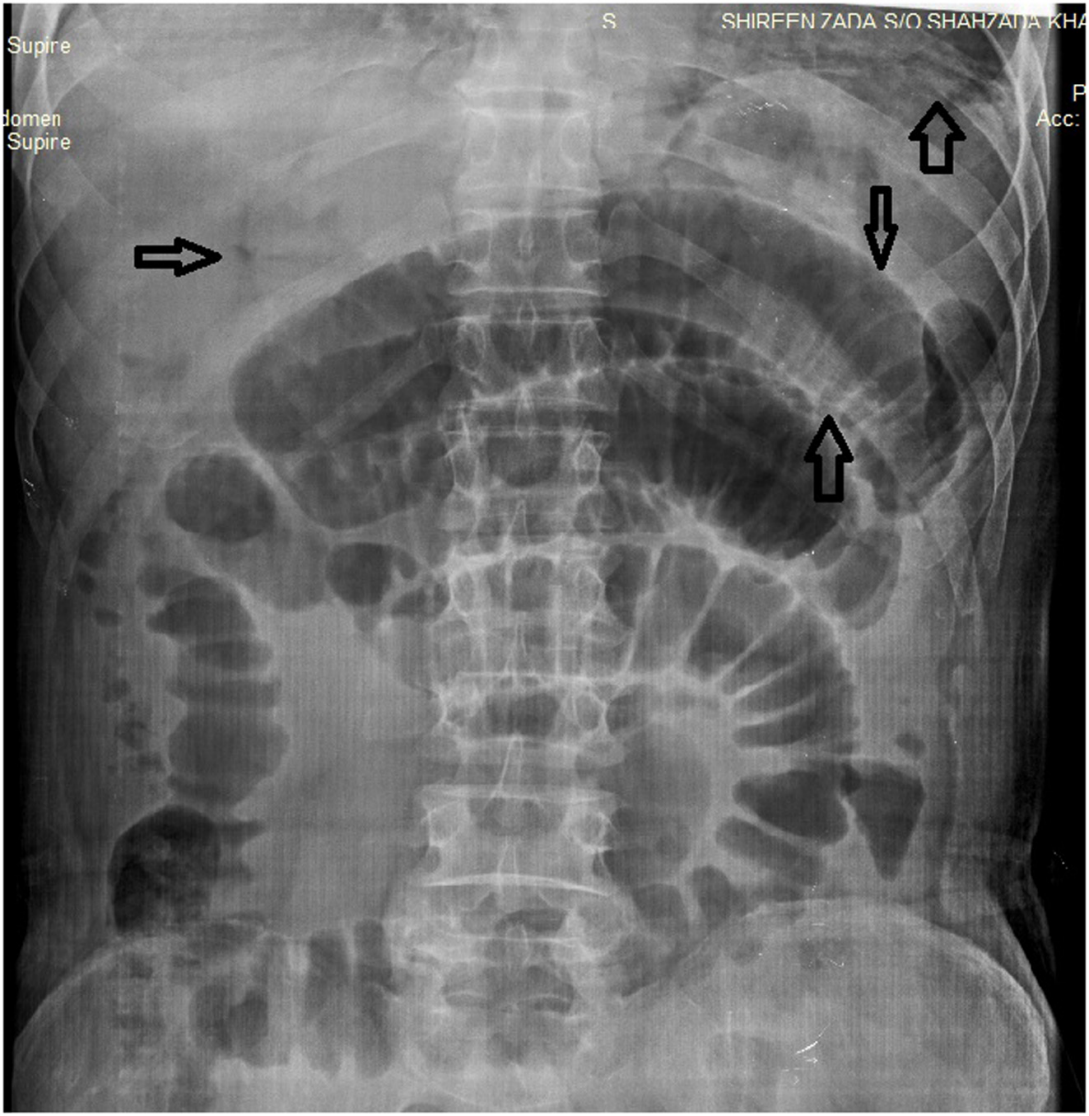
X‐rays of the supine abdomen showing dilated bowel loops, free air, air in the portal vein and Rigler's sign (sign of pneumoperitoneum seen when gas is outlining both sides of the bowel wall). No abnormal calcification was found. Normal densities of soft tissue were identified. No radio‐opaque stone was seen in the KUB area.

**FIGURE 2:**
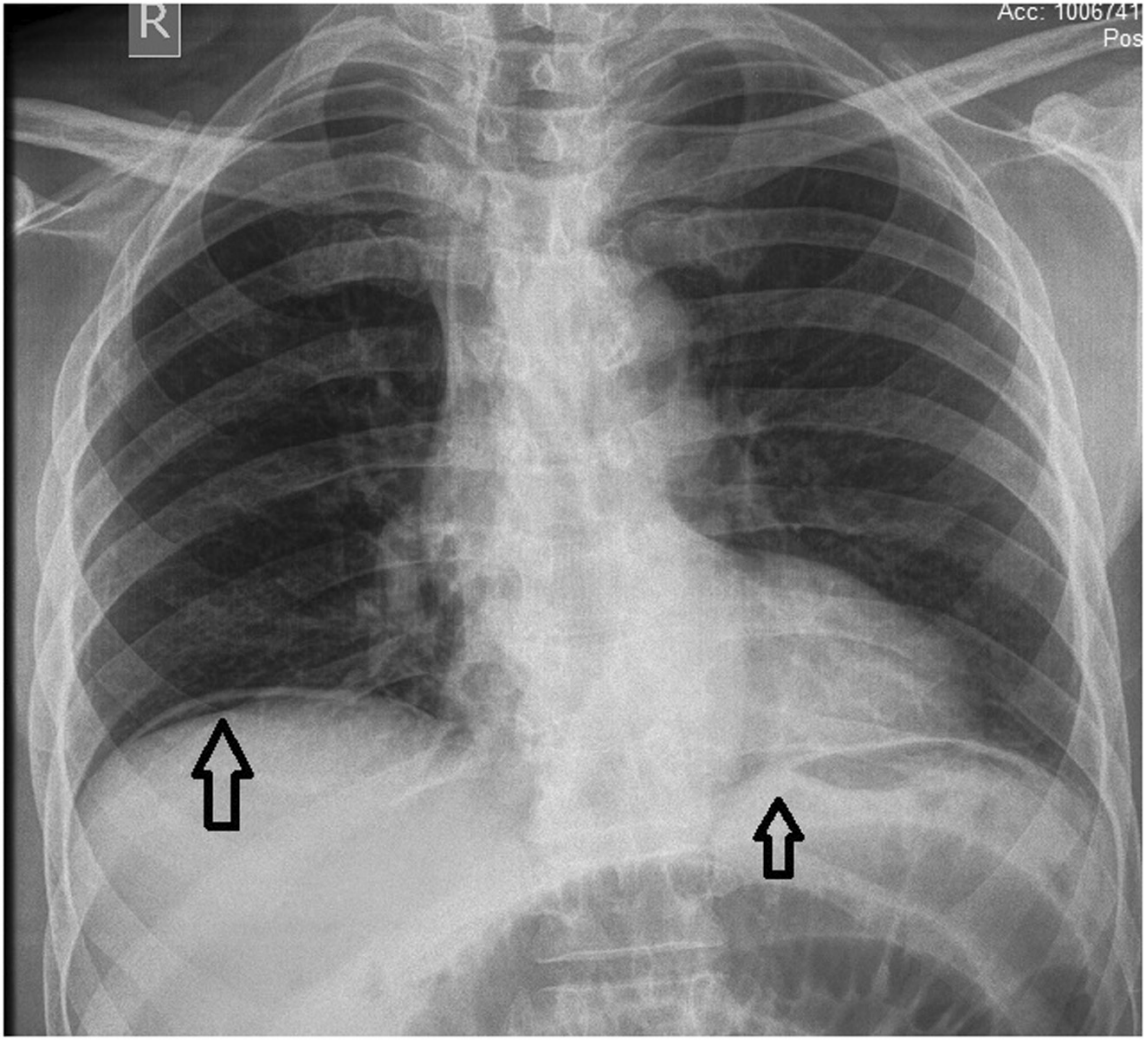
X‐rays showing free air under the right and left domes of the diaphragm. There are no gross signs of consolidation or collapse in either lung field. Hila and mediastinum are normal. Normal costophrenic angles along with normal transverse cardiac diameter were identified. Normal skeletal rib cage symmetry identified.

On general physical examination, the patient was anemic and dehydrated. His blood pressure was 149/76 mm of Hg and his heart rate of 94 beats/min. On examination, the abdomen was firm and swollen (distended), with discomfort in the lower abdomen on palpation. The bowel sounds were audible. Laboratory results showed a total WBC count of 1.9 B/L, hemoglobin 13.7 g/dL, RBC count of 4.12 B/L, platelet count of 122 B/L, absolute neutrophil count of 1520, sodium 138 mEq/L, potassium 3.5 mEq/L, bicarbonate 21 mEq/L, chloride 106 mEq/L, total bilirubin 2.6 mg/dL, direct bilirubin 1.5 mg/dL, and alkaline phosphatase 150 IU/L. X‐RAY imaging of the abdomen in supine posture indicated a few dilated bowel loops and a few air‐fluid levels, suggesting most likely sub‐acute blockage (Figure 1). No abnormal calcification was found. Normal densities of soft tissue were identified. No radio‐opaque stone was seen in the KUB area (Abdominal XRAY in a supine position was performed, which revealed multiple dilated bowel loops, the air in the portal vein, and Rigler's sign). Chest X‐ray in posteroanterior view revealed lucency under the right diaphragm dome, suggesting pneumoperitoneum (Figure 2) (Chest X‐ray in posteroanterior view demonstrated lucency under the right and left domes of the diaphragm, suggesting pneumoperitoneum). There were no gross signs of consolidation or collapse in either lung field. Hila and mediastinum were normal. Normal costophrenic angles along with normal transverse cardiac diameter were identified. Normal skeletal rib cage symmetry was identified. In the context of the aforementioned indicators, acute peritonitis due to intestinal perforation was hypothesized.

**FIGURE 3:**
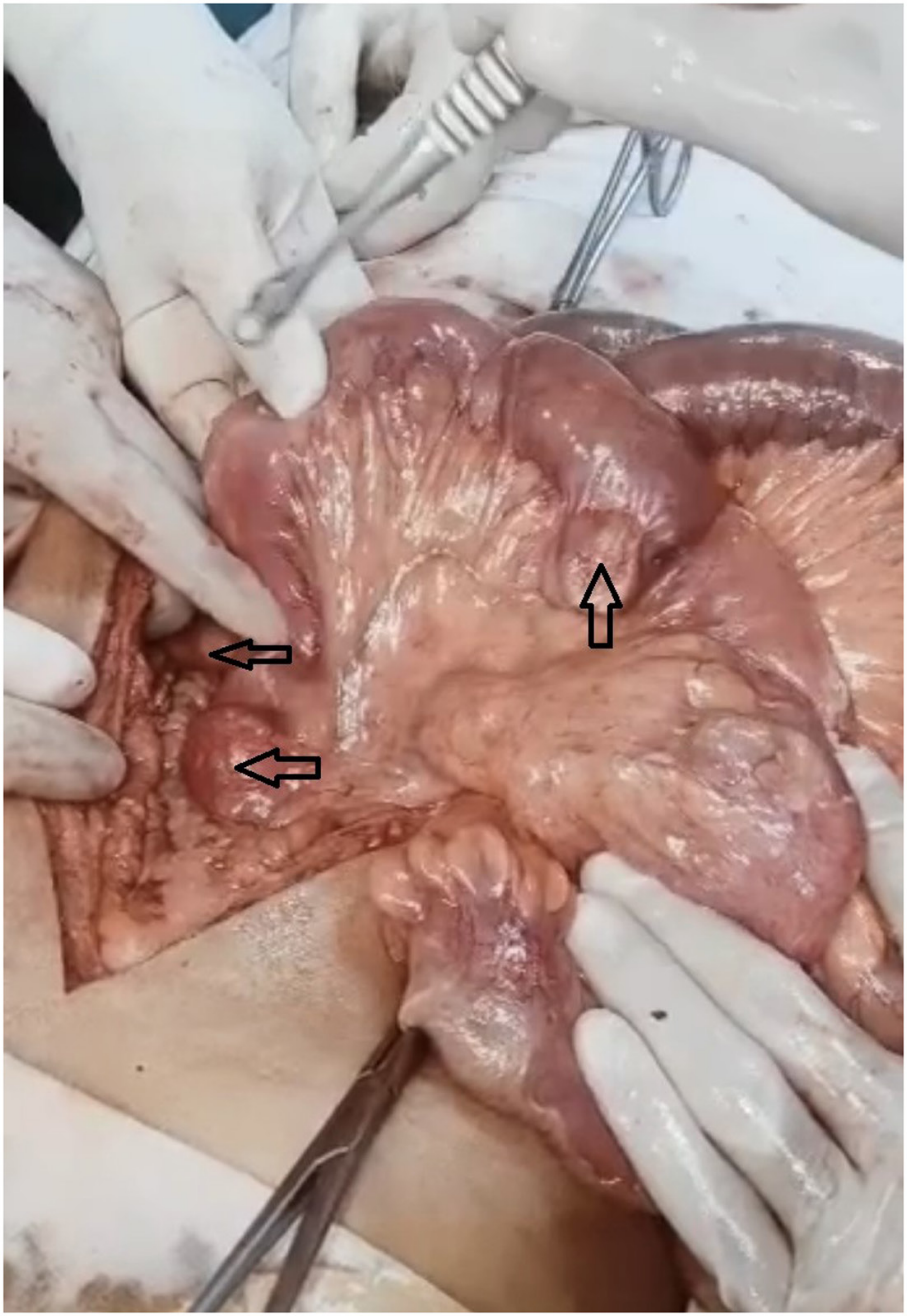
Multiple jejunal diverticula.

Prior to the procedure, a platelet transfusion was performed. Exploratory laparotomy indicated the presence of 1000 mL of ileal contents within the peritoneal cavity. A diverticular perforation (mesenteric boundary) was discovered 1 foot (1 cm) from the ileocecal junction. One diverticulum was 1 foot (1 cm) away from the duodenojejunal (DJ) junction, while the other was 2 feet (2 cm) away from the DJ junction. There was an edematous ileal wall at the site of the perforation. Significant ileal edema necessitated ileal resection and a double barrel ileostomy (Figure 3). The patient was administered ceftriaxone, metronidazole, ketorolac, metoclopramide, and omeprazole postoperatively. The patient recovered without incident and was discharged after 4 days. On discharge, Omeprazole 40 mg once daily (OD) for 14 days, mefenamic acid for pain as needed, metronidazole 400 mg three times a day for 7 days, and Linezolid 600 mg twice daily for 14 days were prescribed, along with milk intake of 200 mL + 4 scoops a day and a high‐protein diet advised.

## 3 DISCUSSION

Most often, diverticula are found in the jejunum, 15% are found in the ileum, and 5% are distributed in both. Jejunal Diverticulosis is mostly a silent disease, and only 29% of patients are symptomatic, while only 10% of patients proceed to develop complications including, obstruction, fistula formation, peritonitis, lower gastrointestinal bleeding, and perforation.^6^ Malabsorption and mesenteric abscess are other complications.^1^ Intestinal obstruction is mainly attributed to adhesions or stenosis, as a result of diverticulitis, as well as intussusception and volvulus associated with the segment containing the diverticulum. Additionally, large stones that are trapped in the diverticula have the tendency to escape or exert stress on local gut wall, resulting in intestinal occlusion.^4^ Perforation of the jejunal diverticula can be caused by diverticulitis, foreign materials and abdominal trauma.^4^ Malabsorption may be explained by irregular bowel movements caused by peristalsis, diverticula enlargement, intestinal stagnation, and bacterial overgrowth.^4^ Acute diverticulitis might result in bleeding because of the erosive effects of the infection. As a result, mesenteric vessels are compromised by mucosal ulcerations, which results in bleeding.^4^

Krishnamurthy et al.^7^ reported intestinal obstruction to be the major clinical presentation of jejunal diverticulosis, with patients presenting with vomiting, abdominal pain, and abdominal distention, which in its most severe form can present with perforation and peritonitis, as did occur in our case. A classical triad consisting of clinical and radiological findings has been described, which includes abdominal pain, anemia, and segmental dilatation in the left upper abdomen or epigastrium visualized on a plain abdominal X‐Ray.^8^ The anemia is attributed to megaloblastic anemia and anemia caused by iron deficiency have both been observed frequently and are frequently related to malabsorptive disorders, steatorrhea, and vitamin deficiencies.^4^ Signs of complications include distention of jejunal bowel loops, multiple air‐fluid levels, and pneumoperitoneum^8^ due to recurrent micro perforations of the diverticula.^4^ While enteroclysis and barium follow‐through are more specific than a plain abdominal X‐ray, there are doubts over their utility in emergency situations.^4^ Computed tomography scan (CT) is a more specific investigation, which demonstrates focal outpouchings on the mesenteric side of the bowel.^4^ For complex instances, laparoscopy becomes a reliable diagnostic method. It also quickly transforms into laparotomy and can serve as a guide to prevent necessary laparotomies. Additionally, by pinpointing the location of the intestinal complication, laparoscopy helps the surgeon choose the best location to make an incision on the abdominal wall, reducing overall on the length of the procedure, the pain encountered thereafter, and the morbidity associated with a larger abdominal incision.^4^ A promising new method for finding small bowel illnesses, wireless capsule endoscopy is mostly employed when there is concealed intestinal bleeding. Despite the relative caution that should be exercised when using capsule endoscopy in patients with isolated small bowel diverticulosis and occult intestinal bleeding, the presence of large diverticula is a relative contraindication because there is a chance that the capsule could become strangulated in small bowel diverticula.^4^

Some patients respond to the brief cessation of enteral nourishment, alleviation of gastrointestinal symptoms provided by a nasogastric tube, and the use of empirical, broad‐spectrum antibiotics, although 8%–30% of patients experience problems necessitating surgical intervention. In the event of a perforation, exploratory laparotomy with resection of the affected intestinal segments and primary anastomosis is necessary. The extent of the intestinal resection depends on the length of the bowel affected, with a more conservative resection of only perforated intestinal segments preferred in cases of involvement of long sections of the bowel, which was the situation in our case.^1^ Other surgical procedures such as invagination of diverticula, closure of perforation with omental patch and diverticulectomy have also been proposed, but they have been associated with high mortality rates.^9^ An operated diverticulum generally has good outcomes, and the postoperative mortality rate is influenced by the time interval between presentation and intervention, the age of the patient, and the type of complications.^10^ If the cause of obstruction was an enterolith, then the stone must be removed by an enterotomy.^4^ Recurrence of diverticula can occur despite intestinal resection as the mechanism of diverticula formation is still patent.^4^

Acquired jejunoileal diverticulosis has a wide range of clinical manifestations. As a result, it may be challenging to diagnose the illness. Up to 90% of patients have been shown to present with symptoms similar to those of irritable bowel syndrome, including intermittent abdominal pain, constipation, and diarrhea. Imaging tests primarily have an atypical appearance without essential diagnostic features and may not correspond with the clinical symptoms.^1^ Contrarily, it can be more challenging to distinguish the diverticula from overlapping loops of small bowel in patients with severe jejunal diverticulosis.^3^ Although a thorough examination of the small bowel is required, it may be challenging to identify jejunal or ileal diverticula during surgery since they are typically concealed in the mesenteric fat.^9^ As a result, the diagnosis is frequently found accidentally after a laparotomy due to difficulties or during a radiographic examination. During a barium swallow, laparotomy, or autopsy, 75% of jejuno‐ileal diverticula are unintentionally detected.^5^ Thus, knowledge of the condition and the numerous forms in which it may manifest is crucial for clinical diagnosis of the disease.^1^ Because perforation is linked to a high mortality rate in up to 40% of patients, a delayed diagnosis can be catastrophic.^5^ Hence, when discovered it should not be discounted as a minor discovery in older individuals who present with unexplained stomach complaints.^1^ Indeed, some authors claim, jejunal diverticulosis need to be routinely taken into account as a potential cause in any patient who presents with inexplicable diarrhea.^3^ The presence of perforated Jejunal diverticulosis should not be discounted in the differential diagnosis of any elderly patient, especially when they present with the classical triad of clinical and radiological findings especially when they have a prior history of constipation, or any pathology that causes chronic raised intra‐abdominal pressure. In such cases of high suspicion and impending peritonitis, a laparoscopy can be a reliable method of diagnosis. However, when presented with symptoms, it is also crucial to include differential diagnosis like neoplasms (with or without perforation), foreign body perforation, traumatic hematoma, medication‐induced ulceration (non‐steroidal anti‐inflammatory drug), and Crohn's disease in our investigation.^5^

## 4 CONCLUSION

To conclude, a high index of suspicion for jejunal diverticulitis should be maintained in patients presenting with acute abdominal symptoms to avoid misdiagnosis and prevent adverse outcomes. Careful clinical assessment coupled with timely radiological evaluation can aid in early detection and appropriate management of this condition, potentially saving lives. Therefore, it is crucial for healthcare providers to remain vigilant and consider jejunal diverticulitis as a possible differential diagnosis in all cases of acute abdomen.

## AUTHOR CONTRIBUTIONS

**Aleeza Jawed:** Writing – original draft; writing – review and editing. **Areesha Jawed:** Writing – original draft; writing – review and editing. **Sandhya Kumari:** Writing – original draft; writing – review and editing. **Omer Ahmed Shaikh:** Writing – original draft; writing – review and editing. **Abdulqadir J. Nashwan:** Writing – original draft; writing – review and editing.

## FUNDING INFORMATION

None.

## CONFLICT OF INTEREST STATEMENT

All authors declared no conflict of interest.

## CONSENT

Written informed consent was obtained from the patient to publish this report in accordance with the journal's patient consent policy.

## Data Availability

All data generated or analyzed during this study are included in this published article.
